# Neurological manifestations of an emerging zoonosis—Human monkeypox virus: A systematic review

**DOI:** 10.1097/MD.0000000000034664

**Published:** 2023-09-01

**Authors:** Sajjad Ahmed Khan, Surya Bahadur Parajuli, Vivek K. Rauniyar

**Affiliations:** a Birat Medical College Teaching Hospital, Morang, Nepal; b Department of Community Medicine, Birat Medical College Teaching Hospital, Morang, Nepal; c Department of Clinical Neurology, Birat Medical College Teaching Hospital, Morang, Nepal.

**Keywords:** brain, human monkeypox virus, monkeypox disease, neurological manifestation

## Abstract

**Background:**

The last few decades have witnessed an appalling rise in several emerging and re-emerging viral and zoonotic outbreaks. Amongst those emerging zoonosis, one of the diseases which is gaining popularity these days and has been declared as public health emergency of international concern by the world health organization, is human monkeypox virus (HMPX). Proper understanding of the clinical spectrum of the disease is of paramount importance for early diagnosis and treatment. In this review, we aimed to study and quantify the neurological manifestations of HMPX virus infection.

**Methods:**

Any study, released prior to April 13, 2023, that reported neurological manifestations in patients infected by HMPX virus were reviewed systematically on PubMed, Scopus, Google Scholar, and Cochrane Library using the PRISMA (Preferred Reporting Items for Systematic review and Meta-Analysis) statement.

**Results:**

Our systematic review included data from 22 eligible studies: 10 cohort studies, 3 cross sectional studies, one retrospective study, 5 case series, and 2 case reports. The most commonly reported neurological manifestations of HMPX were headache (48.84%), myalgia (27.50%), fatigue (17.73%), and photophobia (4.43%). Uncommonly, HMPX can also present with visual deficit (0.57%), seizure (0.34%), encephalitis (0.8%), dizziness (0.34%), encephalomyelitis (0.23%), coma (0.11%), and transverse myelitis (0.11%).

**Discussions:**

Monkeypox virus usually presents with self-limiting painful rash, lymphadenitis, and fever, complications like secondary skin infection, eye problems and pneumonia can be life threatening, carrying a case fatality rate of 1% to 10%. Neurological manifestations are not uncommon and can further add-on to morbidity and mortality.

## 1. Introduction

Monkeypox arises from an orthopoxvirus belonging to the Poxviradea family, which is known to have a complex double-stranded DNA.^[1]^ Clinical manifestation of the disease is similar to but less severe than smallpox, presenting as fever, headache, lymphadenopathy, back pain, myalgia, and skin rash.^[2]^ Rashes usually occurs 1 to 3 days after fever or may coincides with fever sometime, with common sites being; face (97.5%), torso (92.5%), arms (87.5%), legs (85%), genitals (67.5%), scalp (62.5%), palms (55%), soles of feet (50%), mouth (37.5%), and eyes (25%).^[3]^ The disease is usually self-limiting and resolves within 2 to 4 weeks; however, complications like secondary infections, bronchopneumonia, sepsis, encephalitis, and involvement of the cornea with ensuing loss of vision can occur in patients with underlying immunodeficiencies like HIV, other chronic illnesses and absence of previous smallpox vaccination.^[4]^

The first case of human monkeypox virus (HMPX) was confirmed in 1970 in the Democratic Republic of Congo and for decades the disease was confined to the African subcontinent until 2003 when first outbreak outside the African countries was noted.^[5]^ The recent outbreak occurred in the United Kingdom (UK) on May 6 2022 and was subsequently declared as global emergency by the World health organization.^[6]^ Thus, monkeypox virus has become an issue of public health concern but owing to rarity of the disease, little is known about the clinical spectrum of monkeypox. To show what neurological manifestations the disease can present with is the central theme of our study.

## 2. Methods

### 2.1. Search strategy

Data were retrieved from PubMed, Scopus, Cochrane library, and Google scholar for studies published prior to April 13, 2023. The search strategy was based on (monkeypox virus OR human monkeypox virus) AND (neurological OR neurologic OR brain OR CNS OR nervous) AND (manifestation OR symptoms OR presentation).

### 2.2. Eligibility criteria

**Figure 1.**
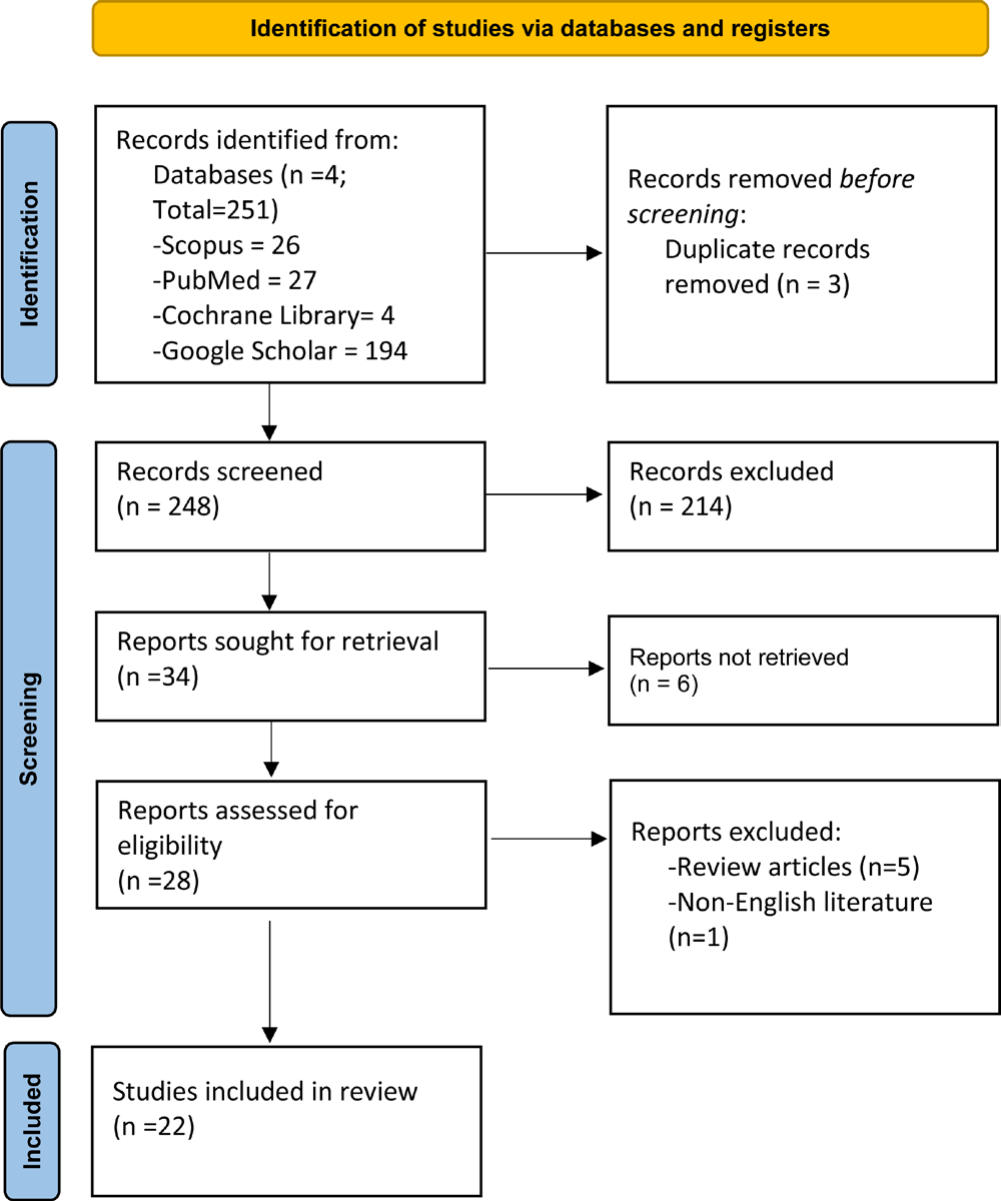
PRISMA flow depicting the flow of information through the different part of systematic review. With the search strategy we adopted, different database was assessed and studies compatible with our inclusion criteria were included in the study.

Applicability of each paper was assessed manually by all authors and articles meeting the inclusion criteria were selected for comprehensive evaluation (Fig. 1).

### 2.3. Inclusion criteria

Articles published in English language dealing with the neurological manifestations of Laboratory confirmed cases (positive IgM antibody and PCR or virus isolation) of monkeypox virus infection were included in the review.

### 2.4. Exclusion criteria

Probable cases and cases without laboratory evidence of monkeypox infection were excluded. We also excluded review articles, hypotheses papers, and papers published in languages other than English.

### 2.5. Data extraction

Data was manually extracted from eligible studies by SAK, SBG, and VKR independently. The following variables were included: first author, year of study, confirmed cases, study title, reported neurological manifestations, and references

### 2.6. Outcome measures

Our outcome was to elucidate the neurological manifestations of HMPX reported in the medical literature.

## 3. Results

### 3.1. Study characteristics

**Figure 2.**
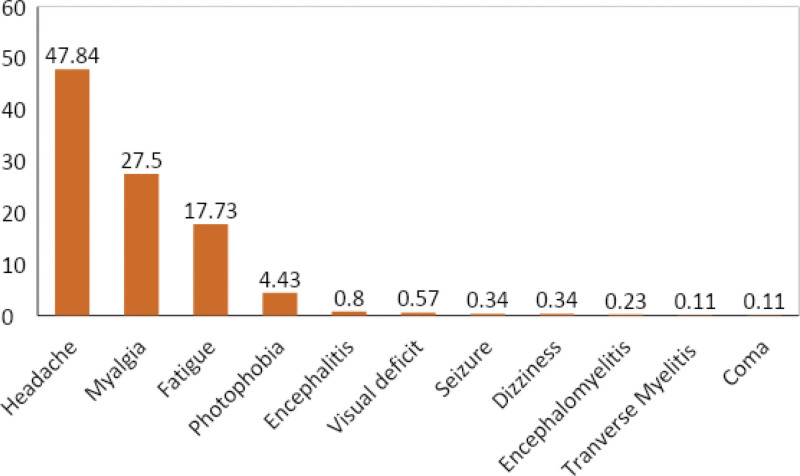
Neurological manifestations of human monkeypox virus expressed as percentage. Rigorous statistical analysis was done to quantify the different neurological manifestations of human monkeypox virus.

We found a total of 251 articles after searching PubMed, Scopus, Google scholar, and Cochrane library database. After excluding duplicates we were left with 248 articles processed for abstract screening. With abstract screening, we excluded 214 articles and ended-up with 34 articles. Among them, 28 articles were assessed for eligibility. Based on our inclusion and exclusion criteria, we excluded 6 articles and finally a total of 22 articles were included in this systematic review (Table 1). Results obtained from the review are as follow (Fig. 2).

**Table 1:** Details of the studies enrolled in this systematic review.

Study	Year	Confirmed cases	Study title	Summary	References
Ongoina et al	2017–2018	40	Clinical course and outcome of human monkeypox in Nigeria	Headache 19, myalgia 25, seizure 1, encephalitis 3, photophobia 9	^[9]^
Ongoina et al	2017	18	The 2017 human monkeypox outbreak in Nigeria – report of outbreak experience and response in the Niger Delta University Teaching Hospital, Bayelsa State, Nigeria	Headache 12, myalgia 5, photophobia 3	^[20]^
Yinka et al	2017–2018	118	Outbreak of human monkeypox in Nigeria in 2017–18: a clinical and epidemiological report	Headache 61, myalgia 42, photophobia 27	^[21]^
Akar et al	2017–2019	165	Descriptive epidemiology of monkeypox in Nigera, September 2017–June 2019	Headache 78	^[22]^
Eseigbe et al	2018	2	Human monkey pox virus infection in Plateau State, north central Nigeria: a report of 2 cases	Headache 2	^[7]^
Hughes et al	2009–2014	134	A tale of 2 viruses: coinfections of monkeypox and varicella zoster virus in the Democratic Republic of Congo.	Headache 99, myalgia 90, fatigue 115	^[23]^
Pittman et al	2007–2011	216	Clinical characterization of human monkeypox infections in the Democratic Republic of the Congo	Headache 49, myalgia 15, dizziness 3, visual deficit 5, fatigue 11	^[24]^
Ježek et al	1980–1985	282	Human monkeypox: clinical features of 282 patients	Encephalitis 1, coma 1	^[8]^
Huhn et al	2003	34	Clinical characteristics of human monkeypox, and risk factors for severe disease	Headache 13, myalgia 19, seizure 1, encephalitis 1	^[10]^
Croft et al	2003	19	Occupational risks during a monkeypox outbreak, Wisconsin, 2003	Headache 13	^[25]^
Reed et al	2003	11	The detection of monkeypox in humans in the Western Hemisphere	Headache 11, myalgia 1	^[26]^
Reynolds et al	2003	37	Clinical manifestations of human monkeypox influenced by route of infection	Headache 32, myalgia 36	^[27]^
Anderson et al	2003	1	A case of severe monkeypox virus disease in an American child: emerging infections and changing professional values	Headache 1, myalgia 1, fatigue 1	^[28]^
Sejvar et al	2003	3	Human monkeypox infection: a family cluster in the Midwestern United States	Headache 2, seizure 1, encephalopathy 1, encephalitis 1	^[11]^
Adler et al	2018–2021	7	Clinical features and management of human monkeypox: a retrospective observational study in the UK	Headache 1	^[29]^
Learned et al	2003	3	Extended interhuman transmission of monkeypox in a hospital community in the Republic of the Congo, 2003	Headache 1, fatigue 2	^[30]^
Reynolds et al	2010	2	Short report: detection of human monkeypox in the Republic of Congo following intensive community education	Headache 1, fatigue 1	^[31]^
Formenty et al	2005	10	Human monkeypox outbreak caused by novel virus belonging to Congo Basin clade, Sudan, 2005	Myalgia 7, headache 5, fatigue 5	^[32]^
Ng et al	2019	1	A case of imported monkeypox in Singapore	Myalgia 1	^[33]^
Charniga et al	2022	21	Estimating the incubation period of monkeypox virus during the 2022 multi-national outbreak	Headache 21, fatigue 21	^[34]^
Pastula et al	2022	1	Two cases of monkeypox-associated encephalomyelitis – Colorado and the District of Columbia, July–August 2022	Encephalomyelitis 2	^[12]^
Cole J et al	2023	1	Monkeypox encephalitis with transverse myelitis in a female patient	Encephalitis 1, transverse myelitis 1	^[13]^

### 3.2. Headache

Headache was reported in 18 out of 22 studies included in this review. We found headache as the leading neurological manifestation of HMPX. Out of 880 participants included in this study, headache was reported to be in 421 participants which correspond to 47.84%. There was a reported case of a 20-year-old male, presented with 1 week history of headache along with fever, pain on swallowing and generalized skin lesions, and similar symptoms were reported in his care-taker but symptoms were less intense than the first case.^[7]^ Ježek et al, in their study of 280 patients, reported that headache (sometimes frontal but usually generalized) usually follows fever; however, in some cases headache preceded the onset of fever.^[8]^

### 3.3. Myalgia

Myalgia was the second most common symptom and was found to be reported in 11 studies and was seen among 242 participants accounting to 27.5%.

### 3.4. Fatigue

In 17.73% of study participants fatigability was noted among a total of 880 patients. Following headache and myalgia, fatigability was the third most prevalent neurological feature of the disease.

### 3.5. Photophobia

Photophobia was reported in a total of 39 patients accounting for 4.43% of the total neurological burden of the disease.

### 3.6. Encephalitis

Encephalitis was reported in a total of 7 (0.8%) patients of the study participants. Ongoina et al reported a female neonate aged 28 days who developed encephalitis (generalized seizures) along with the features of bronchopneumonia (with chest radiographic evidence of lung opacities) and died after 8 days.^[9]^ Huhn et al reported a case of a 6-year old girl who underwent intubation and mechanical ventilation for encephalitis.^[10]^ Sejvar et al reported 3 cases of monkeypox from a family where 2 had mild illness with rash only and one required hospitalization for severe encephalitis, the 2 with mild illness were previously vaccinated for smallpox.^[11]^

### 3.7. Visual deficit

Out of a total of 880 patients, visual deficit was reported among 5 patients corresponding to 0.57%.

### 3.8. Seizure

We did report a total of 3 cases of seizures in the study participants. There was a case of a 43-year-old man with HIV-1 infection who had a CD4 count < 20 cells/μL and died following repeated seizures (suspected to have died from encephalitis).^[9]^

### 3.9. Dizziness

Dizziness, as a neurological symptom, was reported in 3 patients of the study population.

### 3.10. Coma

Coma was reported in one out of 880 patients studies in this review.

### 3.11. Encephalomyelitis

Two cases of encephalomyelitis were reported. Pastula et al reported a case of encephalomyelitis in an immunocompetent gay man in his 30s who was vaccinated for monkeypox but vaccination status against smallpox was uncertain, neurological symptom subsided when treated with 5 sessions of PLEX and was kept under iv Rituximab maintenance.^[12]^

### 3.12. Transverse myelitis

Transverse myelitis along with encephalitis was reported by Cole J et. al. in a female patient. She was treated with methylprednisolone and a single dose of cidofovir.^[13]^

## 4. Discussion

In endemic areas, HMPX is commonly transmitted through zoonosis, while in non-endemic region it is transmitted via human-to-human transmission; though the exact pathophysiology is not known, many cases are reported in men having sex with other men.^[14]^ Though, men having sex with men are particularly at the risk of the transmission of the disease, a case reported from France showed an 18 years old female being infected with the disease after having oral and vaginal sex presenting as ulceronecrotic lesions intravaginally and around her vulva.^[15]^ Apart from sexual contact, it is also believed that the virus can transmit through respiratory secretions and saliva, or through direct contact with the exudate or crust material of the lesion. Viral shedding through feces is another potential source for the transmission of the virus.^[1]^

There are limited studies dealing with the neurological manifestations of HMPX and in this review we have provided a comprehensive evaluation of neurological symptoms prevalent in monkeypox. The spectrum of neurological manifestations extends from general prodromal features seen in any viral illnesses like headache, myalgia, and fatigue to potentially life-threatening conditions like seizure, encephalitis, visual field deficit, and coma. Therefore, there are growing concerns about the long-term effect of monkeypox virus on the CNS and subsequent neurological complications and sequelae. The exact pathophysiology of neuroinvasive and neurotropic nature of the virus is not known but studies carried out in animal models have shown 2 probable ways of transmission; directly through olfactory epithelium after intranasal inoculation and by virus-loaded monocytes in circulation reaching the brain tissue by crossing the blood–brain-barrier.^[16]^

Considering the current surge of monkeypox cases, some next-generation vaccines, including modified vaccinia ankara-bavarian nordic, ACAM2000, LC16m8 vaccine, were approved with limited use in high risk individuals.^[17]^ Similarly, A selected number of antiviral agents, such as tecovirimat and brincidofovir are approved for treating complicated cases of monkeypox infection under randomized controlled trials or an Expanded Access for an Investigational New Drug protocol.^[18]^ However, vaccinations and antivirals are not feasible in every settings and, currently, the majority of mild or uncomplicated monkeypox cases are taken care of with symptomatic treatment and optimizing supportive interventions.^[18]^

Neurological manifestations of the disease can range from mild prodromal features like headache, myalgia, and fatigue to severe and life-threatening complications like coma. There are reported cases of not only patients requiring intensive care but loosing life because of neurological complications of the disease.^[19]^ Thus, neurological complications are rare but can be potentially fatal if not diagnosed and treated on time. Talking about the treatment part, like any other viral illnesses, there is no definitive cure for the disease and supportive management is all that can be offered to patients.^[19]^

## 5. Conclusion

Thus, classically presenting as fever, rash, and lymphadenitis with symptoms similar to but less severe than that of smallpox, a constellation of neurological symptoms like headache, myalgia, fatigue, photophobia can be seen. Uncommonly, HMPX can also present with visual deficit, seizure, encephalitis, dizziness, encephalomyelitis, coma, and transverse myelitis. Though there are vaccinations and antivirals under trials, their use is only limited in high risk cases and the disease is usually self-resolving in mild to moderate cases. Complications like secondary infection, bronchopneumonia, confusion, and eye problems can rarely occur in children and immunocompromised patients and the disease can be fatal in up to 1% to 10% of cases.

## Author contributions

**Conceptualization:** Sajjad Ahmed Khan.

**Methodology:** Sajjad Ahmed Khan.

**Supervision:** Surya Bahadur Parajuli, Vivek K. Rauniyar.

**Writing – original draft:** Sajjad Ahmed Khan.

**Writing – review & editing:** Surya Bahadur Parajuli, Vivek K. Rauniyar.
